# Genomic Degeneration and Reduction in the Fish Pathogen *Mycobacterium shottsii*

**DOI:** 10.1128/spectrum.01158-21

**Published:** 2022-05-17

**Authors:** D. T. Gauthier, J. H. Doss, M. LaGatta, T. Gupta, R. K. Karls, F. D. Quinn

**Affiliations:** a Department of Biological Sciences, Old Dominion University, Norfolk, Virginia, USA; b Department of Infectious Diseases, University of Georgia, Athens, Georgia, USA; c Pathens Incorporated, Athens, Georgia, USA; University of Pittsburgh School of Medicine

**Keywords:** *Mycobacterium shottsii*, genome, mycobacteria

## Abstract

Mycobacterium shottsii is a dysgonic, nonpigmented mycobacterium originally isolated from diseased striped bass (Morone saxatilis) in the Chesapeake Bay, USA. Genomic analysis reveals that M. shottsii is a Mycobacterium ulcerans/Mycobacterium marinum clade (MuMC) member, but unlike the superficially similar M. pseudoshottsii, also isolated from striped bass, it is not an M. ulcerans ecovar, instead belonging to a transitional group of strains basal to proposed “Aronson” and “M” lineages. Although phylogenetically distinct from the human pathogen M. ulcerans, the *M. shottsii* genome shows parallel but nonhomologous genomic degeneration, including massive accumulation of pseudogenes accompanied by proliferation of unique insertion sequences (IS*Mysh*01, IS*Mysh*03), large-scale deletions, and genomic reorganization relative to typical M. marinum strains. Coupled with its observed ecological characteristics and loss of chromogenicity, the genomic structure of *M. shottsii* is suggestive of evolution toward a state of obligate pathogenicity, as observed for other Mycobacterium spp., including M. ulcerans, M. tuberculosis, and M. leprae.

**IMPORTANCE** Morone saxatilis (striped bass) is an ecologically and economically important finfish species on the United States east coast. Mycobacterium shottsii and Mycobacterium pseudoshottsii were originally described in the early 2000s as novel species from outbreaks of visceral and dermal mycobacteriosis in this species. Biochemical and genetic characterization place these species within the Mycobacterium ulcerans/M. marinum clade (MuMC), and *M. pseudoshottsii* has been proposed as an ecovar of M. ulcerans. Here, we describe the complete genome of *M. shottsii*, demonstrating that it is clearly not an M. ulcerans ecovar; however, it has undergone parallel genomic modification suggestive of a transition to obligate pathogenicity. As in M. ulcerans, the *M. shottsii* genome demonstrates widespread pseudogene formation driven by proliferation of insertion sequences, as well as genomic reorganization. This work clarifies the phylogenetic position of *M. shottsii* relative to other MuMC members and provides insight into processes shaping its genomic structure.

## INTRODUCTION

The first reports of striped bass (Morone saxatilis) with ulcerative dermal granulomatous inflammation and visceral granulomatous disease emerged in 1997 and were of concern because of the high economic and ecological value of this finfish (1). Acid-fast bacteria consistent with *Mycobacterium* spp. were observed in lesions, and initial bacteriological surveys of striped bass revealed a considerable variety of mycobacterial isolates. Two dominant isolates from these surveys were described and officially named Mycobacterium shottsii (2) and Mycobacterium pseudoshottsii (3). M. shottsii is a slow-growing (>1 month on solid agar) mycobacterium with rough, nonpigmented colonies and little to no growth above 30°C. Biochemically, *M. shottsii* is negative for arylsulfatase, variable for catalase, negative for pyrazinamidase and Tween hydrolysis, and positive for urease. Both *M. shottsii* and the related M. pseudoshottsii can be differentiated from Mycobacterium marinum by positive niacin production (2, 4). Sequencing of a 458-bp (bp) fragment of the 16S rRNA gene (5) demonstrates ≥99% similarity with M. marinum and Mycobacterium ulcerans and ≥98% similarity with other members of the Mycobacterium tuberculosis clade (6). *M. pseudoshottsii* shares a pMUM megaplasmid, multicopy IS*2404* insertion sequence, and close nucleotide similarity to M. ulcerans-related organisms, and indeed, it has been proposed that this species should be reclassified as an M. ulcerans ecovar (7).

Experimental infection studies in striped bass demonstrate long-term *M. shottsii* persistence (45 weeks) but reduced virulence relative to that of M. marinum (8). In wild fishes, *M. shottsii* is associated with skin lesions and visceral lesions (4, 9; Gauthier, unpublished data), whereas *M. pseudoshottsii* has not been detected in skin lesions. While *M. pseudoshottsii* can be detected in prey items of striped bass, as well as water and sediment in Chesapeake Bay (10), *M. shottsii* has to date been detected only in striped bass and the congeneric white perch (Morone americana) (6, 9).

The inability of *M. shottsii* to grow above 30°C combined with its relatedness to the etiological agent of tuberculosis has piqued interest in its possible use as a mammalian intranasal vaccine vector (11). Further, various aspects of its biology, namely, its nonpigmented phenotype, absence from environmental reservoirs, and dysgonic nature on artificial medium, are suggestive that this bacterium is undergoing adaptation to an obligate lifestyle similar to that seen in other related mycobacteria such as M. tuberculosis, M. ulcerans, and M. leprae. For these reasons, analysis of the complete genome sequence for *M. shottsii* is of interest. Genomic analysis of *M. shottsii* provides insight into the evolution of this bacterium from an M. marinum*-*like ancestor.

## RESULTS AND DISCUSSION

### General characteristics.

**FIG 1:**
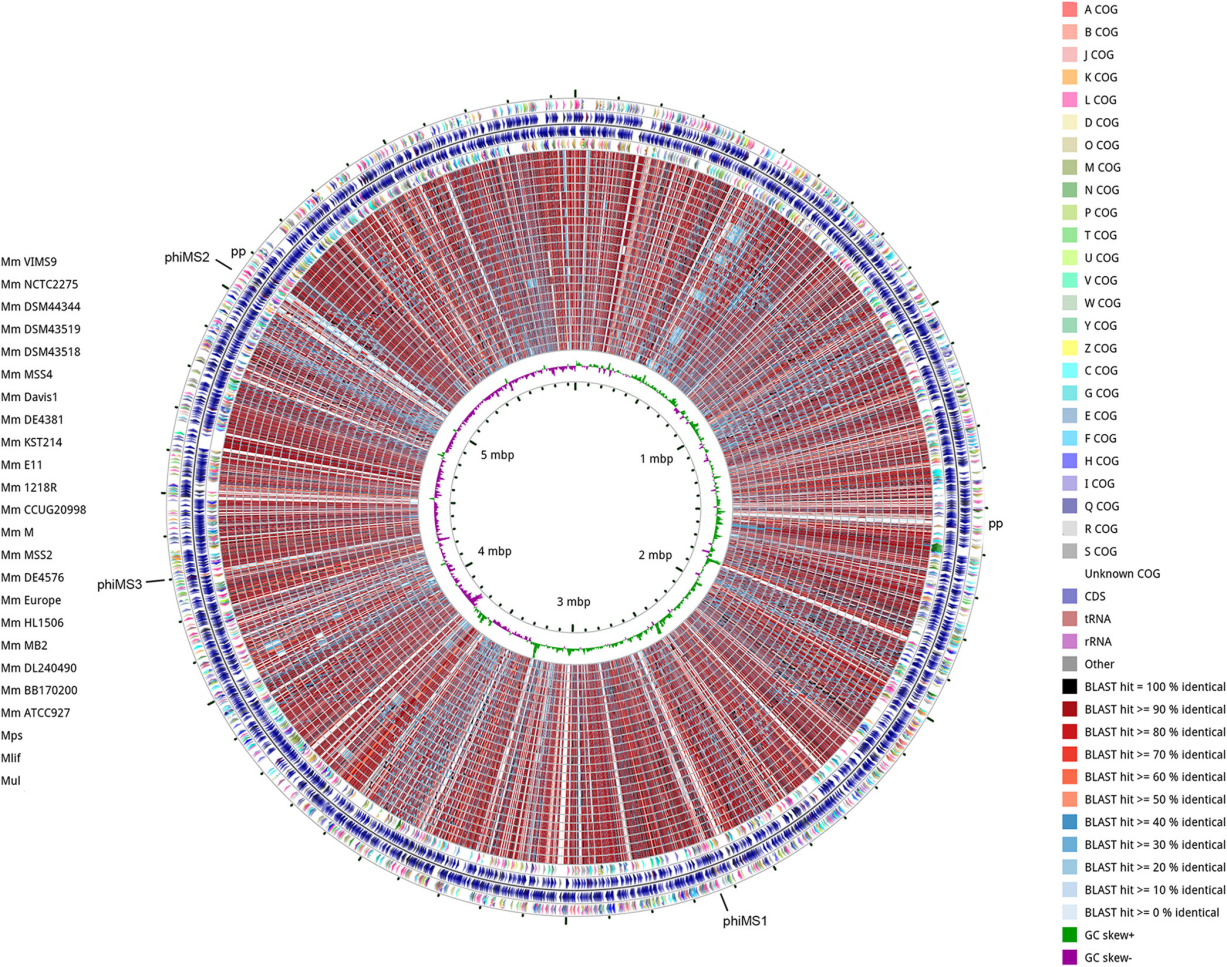
Circular plot of *M. shottsii* M175 genome compared with additional available MuMC genomes via CGView comparison tool (CCT) (49). CDS in forward and reverse directions with COG annotations for *M. shottsii* are indicated in the outer rings, and BLAST-based comparisons to additional genomes are presented as inner rings (default CCT order of highest to lowest similarity to *M. shottsii* reference. The outermost comparison ring begins with M. marinum VIMS9 as indicated in the left legend. Mps, *M. pseudoshottsii*; Mlif, M. ulcerans ecovar Liflandii; Mul, M. ulcerans. Positions of prophages phiMS01-03 and additional putative prophage (pp) elements are annotated on outer ring.

The genome of *M. shottsii* M175 comprises a single 5,956,421-bp chromosome with a G+C% content of 65.5% (Fig. 1). No plasmids were detected during assembly. A total of 4,837 coding sequences and 590 pseudogenes were predicted. One rRNA operon, 48 tRNAs, and one tmRNA were detected. An additional complete genome of *M. shottsii* strain JCM12657 has recently been made available on GenBank (AP022572) but was not discussed in the pertinent article (12). Comparison of AP022572 with the genome presented in this work (CP014860) indicates syntenic assemblies of similar size (5,973,149 and 5,956,421 bp, respectively).

### Insertion sequences.

Insertion sequences (IS) were prevalent in the chromosome (Table 1), including 360 complete copies of 7 individual IS elements, as well as 30 “composite” insertion sequences containing a copy of one of the IS elements contained within another. IS*Mysh*01 was by a large margin the most common insertion sequence in *M. shottsii* and appears unique to this bacterium among the M. ulcerans/M. marinum complex (MuMC). The closest BLAST match for the single transposase coding sequence (CDS) in this IS was to a Mycobacterium thermoresistibile transposase, at 79.9%. Inverted repeats for IS*Mysh*01and the M. thermoresistibile transposase were identical, and both showed structure typical for IS*5*/IS*5* family/group IS elements, including a single open reading frame (ORF), inverted terminal repeats, and 4-base direct repeats (CTAG) (13). IS*Mysh*01 has lower similarity (68.3%) to single-copy transposases from M. marinum strains CCUG 20998 and ATCC 927 and M. tuberculosis group bacteria (IS*Mt*1-family). These IS are annotated with two transposase CDS, consistent with the IS*427* group of the IS*5* family. An additional marginally similar IS*5*/IS*427* transposase is low-multicopy in Rhodococcus opacus (CP009111) and single-copy in *Arthrobacter* sp. (CP040018), suggesting widespread distribution of this IS type among the *Actinomycetales*. The second-highest copy-number insertion sequence IS*Mysh*03 was also unique to *M. shottsii* among the MuMC group, with the closest BLAST matches being to an integrase pseudogene in M. chimaera (86.5%. identity) and to an *Rv3128c*-like protein in Mycobacterium celatum plasmid pCLP (AF312688) (84.4% identity). Family classification of IS*Mysh*03 was not possible. IS*Mysh*04 and 05 were highly similar (≥99%) to insertion sequences IS*Myma*01 and 05, respectively, from M. marinum M. Both of these elements are widespread in MuMC genomes (14). IS*Mysh*02 is highly similar to an IS element present in M. marinum strains CCUG 20998 and ATCC 927, where it is present as a single-copy two-CDS element. In *M. shottsii*, one complete (two-CDS) element is present, but additional copies of the first (15 copies) and second (17 copies) CDS are present at multiple sites in the genome. IS*Mysh*06 and IS*Mysh*07, both present in 4 copies in the *M. shottsii* genome, are present in other Aronson lineage (14) strains, including 1218R, E11, ATCC 927, and CCUG 20998.

**TABLE 1:** Multicopy insertion sequences (IS) in *M. shottsii* M175^*a*^

Name	Length (bp)	Family	No. of copies	BLAST match	BLAST similarity (%)
IS*Mysh*01	930	IS*5*	236	Mycobacterium thermoresistible strain NCTC10409 LT906483	79.9
IS*Mysh*02	2,330	IS*21*	15 (CDS1), 17 (CDS2), 1 (full)	(CDS1) Mycobacterium marinum strain CCUG 20998, (CDS2) Mycobacterium marinum strain CCUG 20998	98.7, 99.1
IS*Mysh*03	1,437	Unc.	97	M. celatum plasmid pCLP, Rv3128c-like protein AF312688	84.4
IS*Mysh*04	1,240	IS*3*	15	M. marinum M IS*Myma*01 CP000854	99.1
IS*Mysh*05	1,836	IS*481*	3	M. marinum M IS*Myma*05 CP000854	99.5
IS*Mysh*06	972	IS*481*	4	M. marinum E11, transposase HG917972	99.7
IS*Mysh*07	1,298	IS*3*	4	M. marinum strain 1218R CP025779	99.3

a Length of entire insertion sequence including inverted repeats (when present) is given in bp. IS families were determined by BLAST searches on ISFinder (https://www-is.biotoul.fr/). Only complete copies of IS elements are tabulated, with the exception of IS*Mysh*02, where only one apparently complete copy was present and all other occurrences were either the first or second CDS of the element. Top BLAST matches were determined by bit-score. Unc, unclassified (ISNCY) (13).

### Phylogenetic relationship.

**FIG 2:**
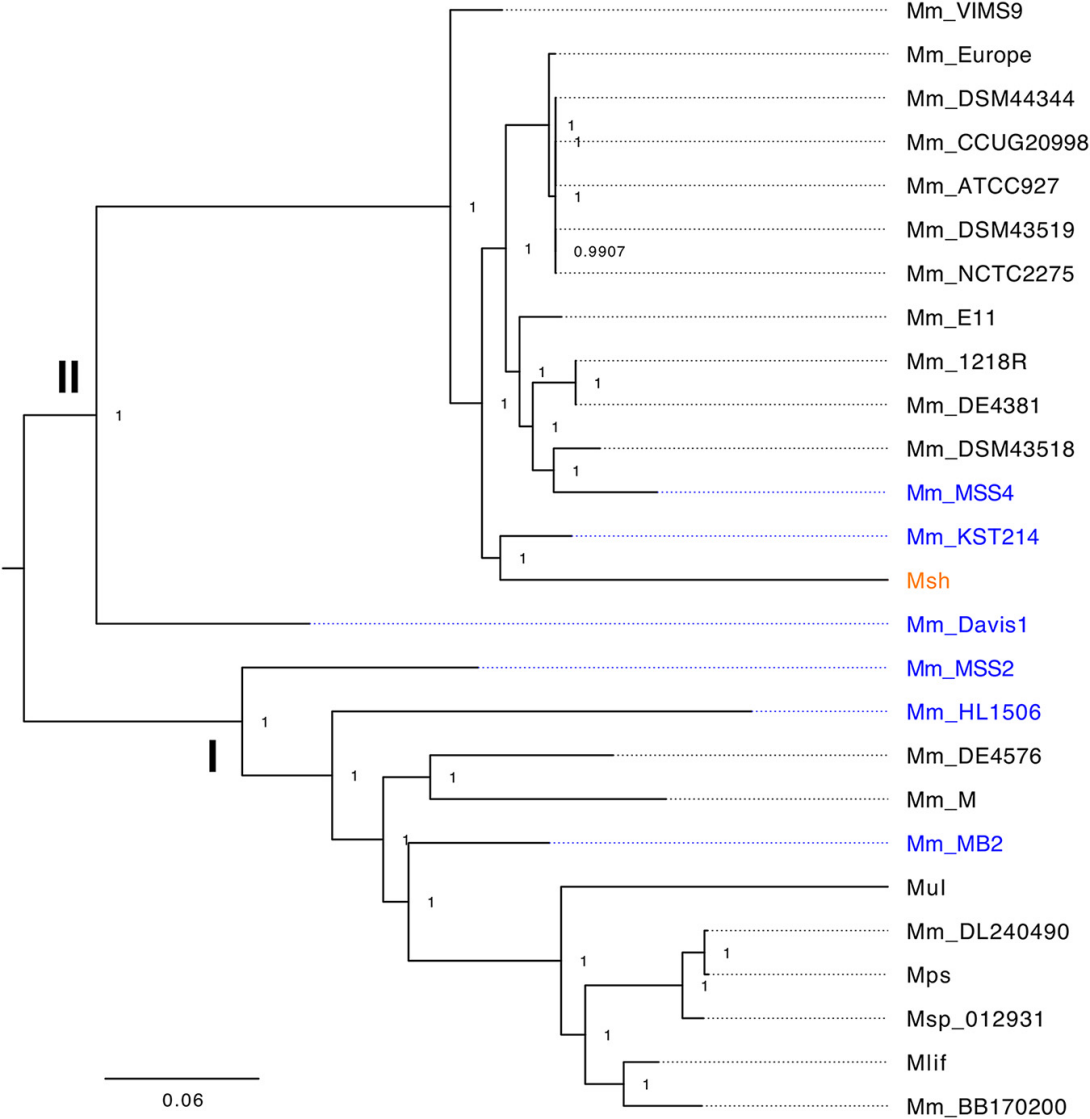
Core SNP-based maximum likelihood phylogeny of *M. shottsii* M175 (Msh; orange) in relation to other complete and draft M. marinum-group genomes in GenBank. Clusters consistent with that of Das et al. (14) are presented as I and II. Msp_012931 (GenBank AOPX01000000) is described in Kurokawa et al. (50). Sources and GenBank numbers for other nodes are Mm_M, M. marinum M, CP000854 (22); Mm_MB2, M. marinum MB2, NZ_ANPM00000000 (51); Mm_Europe, M. marinum *“Europe”* ANPL00000000 (51); ATCC 927, M. marinum ATCC 927, NZ_AP018496 (41); E11, M. marinum E11, NZ_HG917972; Mps, *M. pseudoshottsii*, NZ_BCND01000000; Mul, M. ulcerans Agy99, CP000325 (16); Mlif, M. ulcerans ecovar Liflandii 128FXT, CP003899 (15). Other strains are as described in reference 14. Strains containing complete copies of ESX-2 are shown in blue.

**FIG 3:**
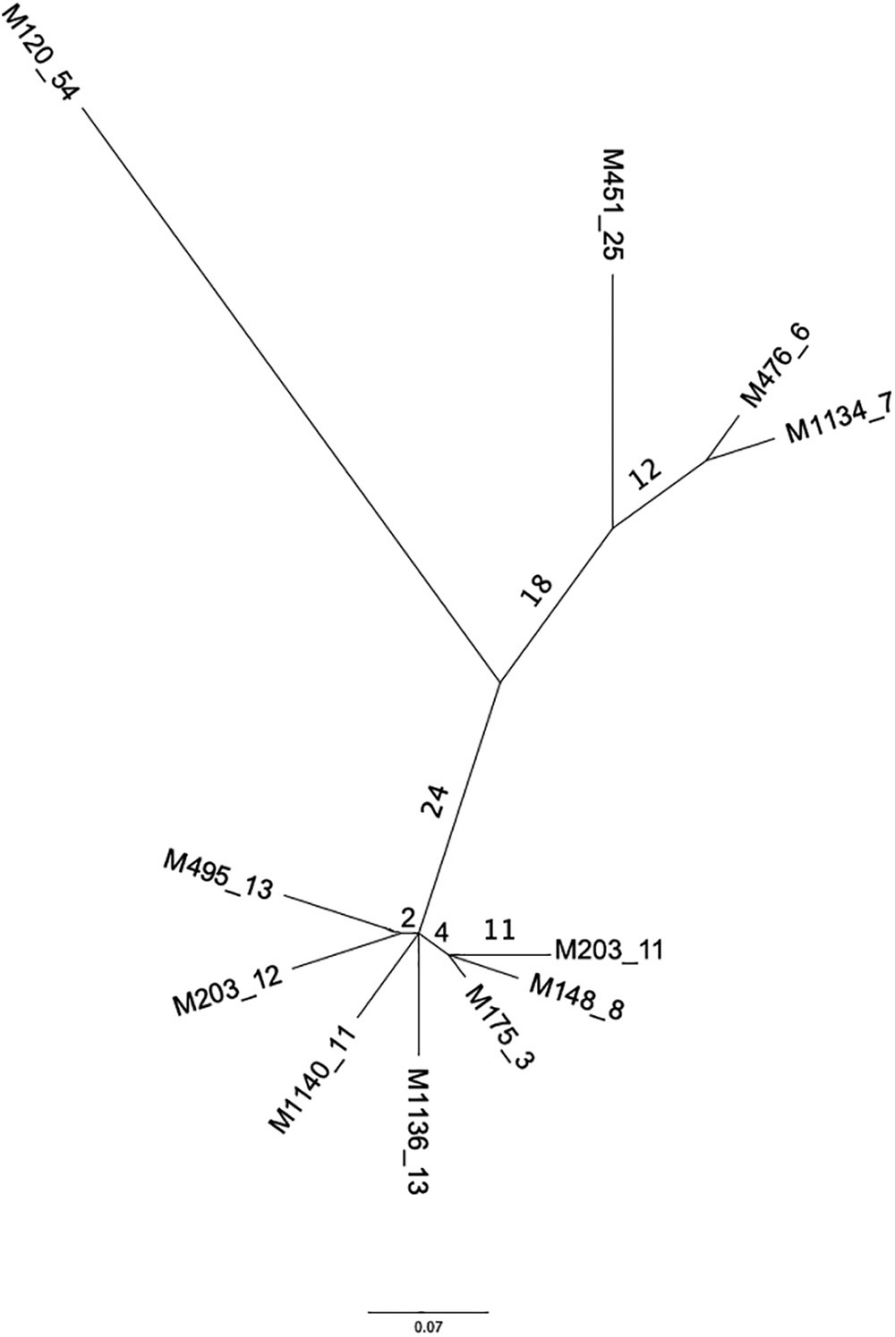
SNP-based core genome phylogeny of *M. shottsii* type strain M175 and additional *M. shottsii* strains isolated from Chesapeake Bay striped bass. Tips include the strain designation followed by underscore and the number of SNPs differentiating that strain. The number of SNPs shared among members of a node are indicated at branches. Scale bar is substitutions per number of SNPs.

Consistent with recent work by Das et al. (14), whole-genome phylogeny of MuMC members demonstrated two clearly separated lineages: cluster I or “M lineage” containing M. marinum M and M. ulcerans group organisms and the clearly distinct cluster II “Aronson lineage” containing M. marinum ATCC 927 and the laboratory strain 1218R (Fig. 2). *M. shottsii* groups with cluster II but occupies a basal divergent branch with the fish isolate KST214 (Fig. 2). This grouping is supported by an average nucleotide identity (ANI) score; *M. shottsii* shares ≥99% ANI with all cluster II strains and ≥98% ANI with all cluster I strains. *M. shottsii* isolates from Chesapeake Bay fishes (*Morone* spp.) were highly similar, with 93 single-nucleotide polymorphisms (SNPs) separating the most distantly related strains. *M. shottsii* M175 belonged to a cluster of nearly clonal strains differing among each other by no more than 19 SNPs (Fig. 3).

### Pseudogenes.

**FIG 4:**
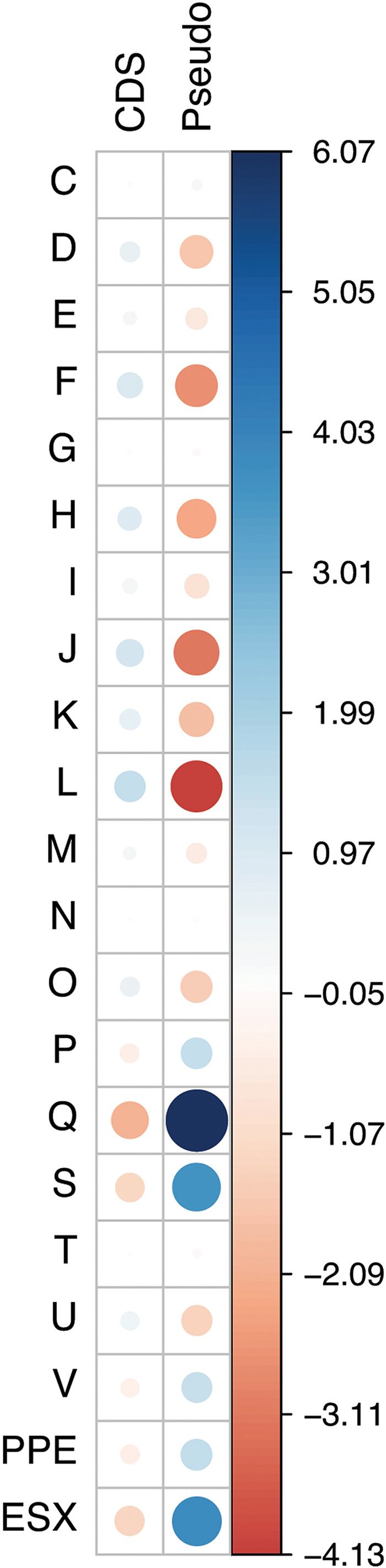
Functional annotation comparison of intact CDS and predicted pseudogenes from *M. shottsii* M175. Clusters of Orthologous Genes (COG) categories are as delineated in the eggNOG database (46) and selectively discussed in the text, with PE/PPE/PE-PGRS-family proteins and ESX loci manually reclassified. Frequencies of functional categories in CDS and pseudogene groups were significantly different (Chi-square analysis, df = 20, *P* < 0.001). Residuals of the Chi-square analysis are plotted as colored circles with the area of circles and density of color representing relative size and direction of residual, as indicated by color scale to right of correlation chart.

A total of 590 pseudogenes were predicted based on comparisons with published M. marinum and M. ulcerans genomes and neighboring BLASTP searches (https://www.ncbi.nlm.nih.gov/genomes/frameshifts/frameshifts.cgi). This number is intermediate between 771 pseudogenes predicted for M. ulcerans Agy99 and 436 for M. ulcerans ecovar Liflandii (15, 16). A minority of pseudogenes for *M. shottsii* (199/590) are also annotated as pseudogenes in M. ulcerans. A substantial number of pseudogenes were created by disruption with insertion sequences (38.8%). Large internal deletions accounted for less than 1% of pseudogenes. Chi-square analysis of the frequency of pseudogenes belonging to different functional categories versus functional (complete) CDS in the *M. shottsii* genome demonstrated a significant difference (Chi-square analysis, df = 20, *P* < 0.001). Examination of residuals from the analysis indicated that secondary metabolite biosynthesis, transport, and catabolism genes (Q) were disproportionately well represented in the pseudogene set, as were ESX-type loci (Fig. 4). Categories representing nucleotide and coenzyme transport and metabolism (F, H), translation (J), and replication, recombination, and repair (L) were negatively correlated with pseudogene formation. Loss of some functions may underlie the extremely low growth rate of this bacterium relative to that of M. marinum and the inability to isolate *M. shottsii* mutants able to grow above 30°C. A more detailed examination of genetic and physiological differences between *M. shottsii* isolates may help define mycobacterial genes responsible for growth rate and temperature restriction.

### Ortholog analysis and regions unique to *M. shottsii*.

**FIG 5:**
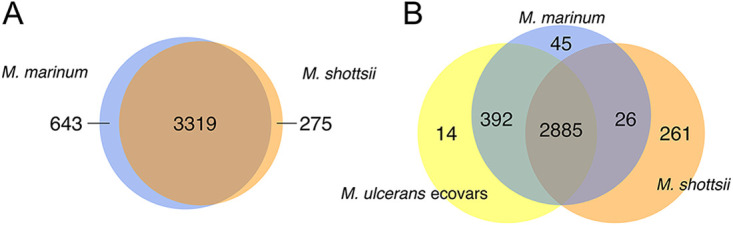
Venn diagram of orthologous and unique proteins between M. marinum strains and *M. shottsii* (A) and among M. marinum strains, *M. shottsii*, and M. ulcerans ecovars (M. ulcerans ecovar Liflandii, M. marinum DL240490, M. marinum BB170200, *M. pseudoshottsii*) (B). Repeat units are collapsed during clustering and are, therefore, reported as a single ortholog.

Ortholog analysis among *M. shottsii* M175 and M. marinum strains excluding M. ulcerans ecovars (Fig. 5A) indicated a core protein set of 3319 CDS, with an additional 275 and 643 CDS unique to *M. shottsii* and M. marinum, respectively. Adding M. ulcerans ecovars (i.e., M. ulcerans ecovar Liflandii, M. marinum DL240490, M. marinum BB170200, and *M. pseudoshottsii*) to the analysis reduced the core protein set to 2,885, and *M. shottsii* retained 261 unique proteins (Fig. 5B). M. ulcerans Agy99 was omitted from this analysis in order to better examine orthology between *M. shottsii* and the more ecologically similar and less-derived members of the M. ulcerans clade. Of 261 CDS determined unique to *M. shottsii* by protein orthology, 8 belonged to two unique partial prophages identified with high confidence via PHASTER analysis: phiMS_2 (*TM48_04614* to *04629*) and phiMS_3 (*TM48_03961* to *03972*). An additional 8 unique CDS in a region (*TM48_04661* to *TM48_04680*) immediately downstream of phiMS_2 also had various degrees of significant BLAST similarity to prophages in other organisms. Additional regions resembling prophage sequences (*TM48_1160-TM48_1162* and *TM48_1426-TM48_1449*) contained 16 CDS unique to *M. shottsii*. Of *M. shottsii-*specific proteins, 121 were annotated as hypothetical proteins, 82 as transposases, and 37 as proline-glutamate (PE)/proline-proline-glutamate (PPE) proteins. A list of proteins representative of each Venn region presented in Fig. 5B is provided in the supplemental material (Table S1). Aside from prophage elements and insertion sequences, *M. shottsii* does not appear to have acquired significant amounts of genetic material from outside sources, and its phenotypic differences from other M. marinum-group organisms appear to stem largely from genomic degeneration and pseudogene accumulation.

### Regions of difference.

A total of 185 deletion region of difference (RD) loci relative to M. marinum M were determined among M. ulcerans, *M. shottsii*, and M. marinum ATCC 927. Thirty-one RDs (16.8%) were present in all three species, including all 10 annotated prophages in M. marinum M and all 4 copies of the IS*Myma*04 insertion sequence. Also included were 2 metabolic loci (M. ulcerans regions of deletion [MURDs] 2 and 73), 4 PPE/PGRS loci (MURDs 7, 25, 32, 50), and 2 regions containing nonribosomal peptide synthase loci (MURDs 2 and 105). Other shared RDs were of unknown function. The majority (83; 44.9%) of RDs were unique to M. ulcerans, and few RDs were conserved between M. ulcerans and M. marinum ATCC 927, including both copies of IS*Myma*05 and a large intermediary metabolism locus that was partially deleted in M. marinum ATCC 972. A larger number of RDs (28; 15.1%) were present in both M. ulcerans and *M. shottsii*; however, these RDs included 7 repeat regions and two copies of IS*Myma*06. Further, few of these RDs demonstrated high reciprocal coverage, with most being small partial deletions contained within a larger deleted region in the opposite species. Twenty-six (14.1%) RDs were unique to *M. shottsii* (Table S2), and 4 (2.1%) were unique to M. marinum ATCC 927. An additional eight RDs (4.3%) were shared between *M. shottsii* and M. marinum ATCC 927 (Table S2). Regions of deletion unique to *M. shottsii* (MSRD) comprised 163 CDS, and the majority (20/26) were flanked by insertion sequences, especially IS*Mysh*03, which was present at half (10/20) of the RD boundaries, despite representing only 27% of insertion sequence copies in the genome. A variety of secondary metabolism loci were represented in *M. shottsii*-specific RDs, including glycosyltransferases and acyl-coenzyme A (coA) dehydrogenase. Three of four copies of acetolactate synthase *ilvB* present in M. marinum M are either pseudogenes or deleted in *M. shottsii.* Acetolactate synthase generates acetolactate from pyruvate and is the first step in the synthesis of the branched-chain amino acids (BCAA) (17). While presence of one intact copy of *ilvB* suggests that *M. shottsii* is not a BCAA autotroph, it does illustrate loss of redundancy in metabolic pathways. Similarly, the gene for DNA ligase B is deleted (MSRD35), as is one copy encoding PpiC peptidyl-prolyl cis-trans isomerase (MSRD27). Peptidyl-prolyl isomerases (cyclophilins) are protein chaperones secreted by M. tuberculosis during intracellular infection and may contribute to virulence by mimicking host cyclophilins (18). Mammalian cell entry (*mce*) operons are virulence factors in M. tuberculosis (19) but may have additional transport roles in saprophytic mycobacteria (20). M. marinum M has seven *mce* operons, while in *M. shottsii*, two are deleted as MSRD8 and MSRD99/MURD132 and a third (MSRD82) is deleted for the associated YrbE transporter permeases (21).

### Respiratory potential.

Mycobacterium shottsii possesses requisite pathways for aerobic respiration, including intact glycolysis and pentose phosphate pathways. Glyoxylate shunt enzymes isocitrate lyase and malate synthase are intact, and therefore *M. shottsii*, like other members of the MuMC, should be able to utilize 2-carbon compounds for synthesis of carbohydrates. A complete tricarboxylic acid (TCA) cycle is present. Beta-oxidation pathways are predicted to be present and functional, and like M. marinum M, a high level of gene duplication is observed at most levels of the pathway. Forty FadD (acyl-CoA synthase) paralogs are predicted, as are 25 EchA (enoyl-coA oxidase) and 12 FadA (3-ketoacyl-CoA thiolase) paralogs. The alternate anaerobic respiratory pathway genes annotated as nitrate reductase and formate dehydrogenase in M. marinum M (22) are intact, whereas they are pseudogenes in M. ulcerans. Unlike M. marinum M or M. ulcerans, the *hyc* operon encoding a formate hydrogenlyase system is disrupted by an *hycC* pseudogene in *M. shottsii*, potentially suggesting reduced flexibility in mixed-acid fermentative respiration (23).

### Cell wall lipids.

All requisite CDS for type I and type II fatty acid synthase pathways are intact in *M. shottsii*; therefore, production of general cell wall mycolic acids is predicted to be as in other *Mycobacterium* spp. The modular type I polyketide synthase operon, *ppsA-E*, is intact in *M. shottsii* M175 and other sequenced strains. This operon in mycobacteria is involved in production of phenolphthiocerol, which is further esterified with mycocerosic acids to form phthiocerol dimycocerosates (PDIM) and phenolic glycolipids (PGL) (24, 25). These cell wall lipids are important virulence factors in tuberculous mycobacteria and are reported to be necessary for virulence in M. marinum (26). Other polyketide synthase loci are consistent with M. marinum M, including *pks12*, the phenolphthiocerol-producing *pks15/1* locus, and *pks13*. The *pks10*,*7*,*8*,*9*,*11* locus, present in M. marinum M and essential for virulence in M. tuberculosis (27), is potentially disrupted in *M. shottsii* (*TM48_02746* to *TM48_02752*), as it is in M. ulcerans Agy99. The gene disruptions to this locus in *M. shottsii* and M. ulcerans differ in location, and an IS5-family transposase is present in *M. shottsii*. Other PKS loci described in M. marinum M (i.e., *MMAR_RS05840* and *MMAR_RS18930* to *MMAR_RS18950*) are not present in either *M. shottsii* or M. ulcerans. The phytoene dehydrogenase *crtI*, one of five genes involved in biosynthesis of the carotenoid pigment isorenieratene, responsible for orange pigmentation in chromogenic mycobacteria (28), is a pseudogene (premature stop) in *M. shottsii* as it is in M. ulcerans and is likely responsible at least in part for the nonpigmented phenotype of *M. shottsii.*

### PE/PPE repertoire.

Ninety-one and 79 CDS, respectively, are identified as PE and PPE family proteins in *M. shottsii*, representing a total of 7.3% of coding capacity. The number of PE/PPE proteins in *M. shottsii* is intermediate to that in M. marinum M (175 PE/106 PPE, 9.1% coding capacity) and M. ulcerans Agy99 (70 PE/46 PPE, 3.8%). PE/PPE proteins are hypothesized to have proliferated among M. tuberculosis and related species, including the MuMC (29). An additional 28 PE/PPE sequences are identified as pseudogenes in *M. shottsii*, which still falls well short of the total in M. marinum M. Given the relatively basal phylogenetic position of *M. shottsii* among the MuMC, it appears that PE/PPE proliferation may still be under way in M. marinum, while contracting significantly in specializing clades such as M. ulcerans and its ecovars.

### ESX loci.

ESX loci encode type VII secretory systems necessary for secretion of the M. tuberculosis early secretory antigenic target (ESAT-6/EsxA) family proteins and other proteins from mycobacteria. Pathogenic mycobacteria encode as many as five type VII secretion systems, ESX-1 to ESX-5; ESX-1, ESX-3, and ESX-5 are essential for bacterial viability or virulence (30–32). ESX-1 is required for escape of M. tuberculosis and M. marinum from phagosomes in macrophages (33). Deletion of the ESX-1 locus (RD1) in M. bovis bacillus Calmette-Guérin (BCG) is the primary attenuator of the strain (34). The genome of M. marinum M contains 29 *esx* genes in at least 5 loci (ESX 1 to 5) (22). ESX-1 is partially duplicated in M. marinum (ESX-6), resulting in nearly identical copies of *esxA* and *esxB* secretion system genes. *M. shottsii* has one copy of *esxA* and *esxB* loci (*TM48_00416* to *TM48_00417*). Relative to M. marinum M, the ESX-1 locus of *M. shottsii* (*TM48_00412* to *TM48_00427*) is disrupted downstream of *eccD1* (*MMAR_RS27385*) by several insertions of IS*Mysh*01 and IS*Mysh*02 and appears to have undergone genomic reorganization with the remainder of the locus (*espK-mycP1*) found at *TM48_05472* to *TM48_05476*. At least one hypothetical protein (*MMAR_RS27390*) has been disrupted by frameshift in this region. This reorganization is not present in M. marinum ATCC 927 or M. ulcerans, and its effect on ESX-1 function in *M. shottsii* remains to be resolved.

**FIG 6:**
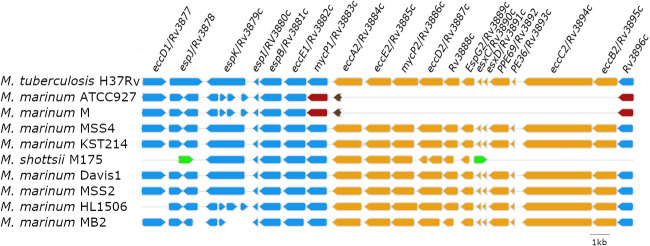
Synteny of ESX-2 locus (orange) of M. tuberculosis H37Rv (*Rv3884c* to *Rv3895c*) relative to *M. shottsii* M175 and several M. marinum strains where ESX-2 is present. M. marinum strains ATCC 927 and M with missing ESX-2 locus are shown for comparison. Flanking CDS of M. marinum ATCC 927 (*MMRN_RS28480* to *MMRN_RS28490*) and M (*MMAR_RS27415* to *MMAR_RS27425*) are shown in red. Partial deletion of *eccA2* ortholog (22) is shown in brown (*MMRN_RS28481* and *MMAR_RS27420*, respectively). Direction of arrows represents strand of gene; genes are drawn to scale. *M. shottsii* M175 partial ESX-2 locus is flanked by IS*Mysh*01 elements (green).

*M. shottsii* ESX-2 has a complete *eccA2* locus, unlike M. marinum M; however, *esxC* and *esxD* loci are absent as they are in M. marinum M, M. ulcerans, and M. ulcerans ecovar Liflandii. Examination of other available M. marinum genomes, however, reveals that complete ESX-2 operons such as those found in M. tuberculosis are present in several strains (MSS2, MSS4, Davis1, KST214, HL1506, MB2), and a partial operon flanked by IS*Mysh*01 repeats is present in *M. shottsii* (Fig. 6). The presence of the ESX-2 locus in *M. shottsii* and additional sequenced M. marinum strains somewhat clarifies the phylogenetic relationships within the MuMC, which can have variable topology depending on which genomic-level data are used to generate trees (14). A complete ESX-2 locus is present in complete genomes of all strains with intermediate placement to Aronson and M lineages (i.e., MSS4, KST214, Davis1, MSS2, HL1506). This character is not monophyletic, however, and is also present in strain MB2, which is interior to the M lineage. Possible explanations for this pattern include horizontal transfer, independent deletion events, or incomplete lineage sorting; however, all other MuMC members have an identical deletion of 11 CDS in this region with a partial deletion of the *eccA2* locus (Fig. 6), strongly suggesting incomplete lineage sorting as an explanation. The presence of ESX-2 appears to be an ancestral character for the MuMC, and those strains possessing it, with exceptions, appear to form a basal transitional group intermediate to the M and Aronson lineages. The role of ESX-2 in survival and virulence of mycobacteria is less well understood than that of other ESX systems (35), but its absence in most members of the MuMC indicates that any attenuation of virulence is compensated by the other ESX loci.

The *M. shottsii* ESX-3 locus appears intact, as does that of ESX-5. ESX-3 functions in mycobactin-mediated iron acquisition in slow- (typified by M. tuberculosis) and fast-growing (such as Mycobacterium smegmatis) mycobacteria (36, 37). ESX-5 is present only in slow-growing mycobacteria and functions in secretion of a large number of PE and PPE multigene family proteins and outer membrane permeability (31, 38). Despite the fact that ESX-4 is considered to be the most ancestral ESX-system based on its presence in other actinobacteria (29), the *eccB* gene in ESX-4 is a pseudogene in *M. shottsii* (*TM48_02015*), disrupted by IS*Mysh*01.

### Conclusions.

The Mycobacterium marinum*-*related pathogens Mycobacterium shottsii and Mycobacterium pseudoshottsii were described around the same time (early 2000s) from the same fish species (striped bass, *Morone saxatilis*) and are closely similar at major gene loci used for multilocus sequence typing (MLST), as expected for members of the MuMC. Further genomic-level work was performed on *M. pseudoshottsii*, demonstrating it to be part of the M. ulcerans clade, and it underwent genomic modifications similar to those of M. ulcerans, including acquisition of the pMUM megaplasmid and highly multicopy insertion sequence IS*2404* (7). The present work, while confirming the membership of *M. shottsii* in the MuMC, demonstrates that this bacterium does not possess the genomic hallmarks of the M. ulcerans clade. Previous work has suggested that *M. shottsii*, like M. ulcerans, is undergoing niche restriction compared to more generalist environmental M. marinum strains (10), and the complete genome demonstrates modifications that parallel, but are distinct from, those of M. ulcerans, such as accumulation of insertion sequences, deleted regions, and pseudogenes.

## MATERIALS AND METHODS

### Bacterial culture.

Mycobacterium shottsii strain M175 was cultured in Middlebrook 7H9 broth (Difco) supplemented with 10% albumin-dextrose-sodium chloride (ADS) (39), 0.5% glycerol, and 0.25% Tween 80 at 25°C, 5% CO_2_, with gentle shaking (60 rpm). Mycobacterium marinum ATCC 927 was obtained from the American Type Culture Collection and grown in Middlebrook 7H9 broth supplemented with oleic acid/albumin/dextrose/catalase (OADC) and 0.05% Tween 80 at 25°C with gentle shaking.

### Genome optical mapping.

*M. shottsii* cells were grown in 7H9 ADS plus 0.5% glycerol plus 0.25% Tween 80 at 25°C to an optical density at 600 nm (OD_600_) of 0.8 to 1.0, treated with cycloserine (1 mg/mL) and carbenicillin (0.1 mg/mL), and then incubated 3 additional days. Cells were harvested by centrifugation (2,800 × *g*, 5 min) at room temperature, washed with spheroplasting buffer (8.4 mM citric acid, 29 mM sodium hydrogen phosphate, 50 mM EDTA, and 0.1% wt/vol Tween 80), and suspended in 1 mL of the same buffer. Bacteria were counted using a Petroff-Hausser chamber, and suspensions of 2 × 10^9^ and 4 × 10^9^ cells/mL were prepared by dilution with spheroplasting buffer. Aliquots of 0.5 mL were then mixed with an equal volume of molten (55°C) 1.5% SeaPlaque GTG agarose (Lonza, Inc.), dissolved in 50 mM EDTA, and then pipetted into 50-well disposable plug molds (Bio-Rad, Inc.) and incubated 90 min at 4°C.

Plugs of agarose-embedded bacterial cells were incubated in lysis buffer 1 (lysozyme [1 mg/mL] and DNase-free RNase A [0.02 mg/mL] dissolved in 6 mM Tris-HCl [pH 7.6], 1 M NaCl, 100 mM EDTA [pH 8.0], 0.5% Brij 35, 0.2% deoxycholic acid, and 0.5% N-lauroyl sarcosine) at 37°C for 24 h and then incubated in lysis buffer 2 (proteinase K [1 mg/mL] dissolved in 0.5 M EDTA [pH 9.0 to 9.5] and 1% N-lauroyl sarcosine) at 50°C for 48 h. The plugs were washed five times with TE buffer by gentle inversion to remove detergent residue and shipped in Tris-EDTA (TE) buffer to Opgen (Gaithersburg, MD). Genome mapping of restriction endonuclease NheI sites was performed at Opgen using previously described methodology (40).

### Illumina and PacBio sequencing.

*M. shottsii* M175 was cultured as described above in 50 mL of medium and harvested by centrifugation (2,800 × *g*, 15 min). Cells were washed with TE buffer and extracted with chloroform methanol (2:1), and phases were separated by centrifugation (2,800 × *g*, 15 min). The cells were recovered and suspended in 1 mL disruption buffer (4 M guanidine thiocyanate, 0.025 M sodium citrate, 0.5% Sarkosyl, and 100 mM 2-mercaptoethanol) with medium vortexing for 1 min, and cell debris was sedimented by centrifugation (4,000 × *g*, 15 min). Supernatant was wadded to a 2 mL heavy phase-lock gel tube (5 Prime, Inc.) and extracted with an equal volume of phenol-chloroform/isoamyl alcohol (25:24:1) by inversion. Phases were separated by centrifugation (16,000 × *g*, 5 min). The aqueous phase was extracted a second time with phenol-chloroform/isoamyl alcohol (25:24:1), and particulates were removed by centrifugation (4,000 × *g*, 5 min). The DNA was precipitated with 0.3 M sodium acetate (pH 5.2) and an equal volume isopropanol, pelleted by centrifugation (16,000 × *g*, 15 min, 4°C), washed with cold 70% ethanol, dried by evaporation, dissolved in nuclease-free water over several days, and treated with DNase-free RNase A. Mycobacterium shottsii M175 DNA was sequenced using MiSeq PE250 platform at the University of Georgia Genomics Center. Other *M. shottsii* strains and M. marinum ATCC 927 were extracted using the DNeasy extraction kit (Qiagen, Valencia, CA) preceded by beadmilling with 0.1-mm Si-Zi beads for 40 s at 5,000 rpm. DNA quantity and quality were determined using NanoDrop. *M. shottsii* strains were sequenced with Illumina PE250 chemistry at the UT Austin Genomic Sequencing and Analysis Facility to a depth of at least 50×. *S*equencing using the PacBio Sequel system was performed at the Centers for Disease Control and Prevention (*M. shottsii* M175) and Washington State University (M. marinum ATCC 927). PacBio sequencing of *M. shottsii* M175 using 3 single-molecule real-time (SMRT) cells (10 kb prep) resulted in 499,283 reads, total read length of 1,058,725,898 bases, mean read length of 2,120 bases, and 153× genome coverage. After assembly with HGAP3, 14 contigs resulted, and 13 were spurious (<20× coverage). After sequences were trimmed and requivered, a genome of 5,956,408 bp was obtained. The genome was polished with Pilon (v1.23) under defaults using Illumina reads, and a final genome size of 5,956,421 bp was obtained. PacBio sequencing of M. marinum ATCC 927 resulted in 137,098 reads, total read length of 1,210,762,761 bp, and ~144× coverage of two contigs of the chromosome (6,496,954 bp) and a plasmid (141,717 bp). This assembly of M. marinum ATCC 927 was used for analyses described in this work that occurred before the completed ATCC 927 genome was made available by a separate group (NZ_AP018496) (41). Comparison of these assemblies indicated that they were syntenic and consistent in size, although the plasmid sequenced in the present work was longer than that reported in reference 41 (127,402 bp).

Celera whole-genome sequencing (WGS) (42) was used to assemble Illumina data from non-M175 *M. shottsii* strains. Mapsolver software was used to compare the PacBio *M. shottsii* genome to the optical map (Fig. S1). *M. shottsii* M175 and M. marinum ATCC 927 genomes were annotated using Prokka (43) with manual curation. Insertion sequences and repeat elements were detected with ISQuest (44). Pseudogenes were manually curated using guidance from Prokka annotations and comparison with published M. marinum and M. ulcerans ecovar genomes as well as neighboring BLASTP searches (https://www.ncbi.nlm.nih.gov/genomes/frameshifts/frameshifts.cgi). For purposes of comparative analyses, genomes were reannotated with Prokka from original .fasta files.

### Bioinformatic analysis.

Core and total genomic single-nucleotide polymorphism (SNP) alignments were generated using kSNP v.3.021 (45). Phylogenetic trees under maximum likelihood were generated with kSNP. Functional ortholog analysis was performed using eggnog 4.5 (46). For pseudogene functional categorization, the predicted function of the orthologous coding sequences (CDS) from either M. marinum M or M. marinum ATCC 927 is reported. Ortholog analysis was performed with ProteinOrtho (v. 6.0.30) using default settings, -singles option to identify unique proteins and the -blastself option to collapse multicopy CDS (47). Publicly available draft and complete genomes of MuMC members were included in the analysis as for the phylogenetic tree (Fig. 1), with the exception of M. ulcerans Agy99. Venn diagrams were produced in Rstudio (R version 4.1.1) with the VennDiagram package. Prophages were identified using PHASTER (PHAge Search Tool—Enhanced Release) (48). The list of putative prophage sequences was curated manually. To be considered a likely prophage, a region was required to fulfill at least two of the following three criteria: (i) identified as a prophage by PHASTER, (ii) at least one match to a known phage gene of one of the classes integrase, tapemeasure, capsid, tail, lysin, portal protein, excisionase, or terminase (e value less than 1e−4), and (iii) shares an insertion site with a known M. marinum prophage. Deletion regions of difference (RDs) were detected in *M. shottsii* or M. marinum ATCC 927 relative to reference genome M. marinum M (Table S2). A custom Perl script (https://github.com/DGauthierLab/misc_scripts) was used to detect RDs of ≥750 bp in length with <5× coverage. RDs from *M. shottsii* and M. marinum were compared for sequence conservation with M. ulcerans regions of difference (MURDs) and to the M. marinum M genome. Regions with <50% overlap were considered unique to a particular organism. In some cases, multiple RDs from an organism were contained within a larger RD from another; in these cases, the larger locus was characterized, and the smaller RD was subsumed within.

### Data availability.

The complete *M. shottsii* M175 genome sequence is available from NCBI GenBank, accession number CP014860. The complete M. marinum ATCC 927 genome was recently made available (41) (GenBank NZ_AP018496) and is syntenic and consistent with the ATCC 927 sequence produced in this work. Sequence reads for *M. shottsii* strains other than M175 are available from the NCBI Sequence Read Archive (PRJNA816683).
