# Supplementary material for: Genomic Degeneration and Reduction in the Fish Pathogen Mycobacterium shottsii
Source: Microbiol Spectr. 2022 May 17;10(3):e01158-21. doi: 10.1128/spectrum.01158-21 (PMC9241763; doi:10.1128/spectrum.01158-21)
Supplement: SUPPLEMENTAL FILE 2 — Supplemental material. Download spectrum.01158-21-s002.pdf, PDF file, 0.2 MB [file spectrum.01158-21-s002.pdf]

Supplemental Table S2. Unique *M. shottsii* M175 regions of deletion (MSRD)

RD	Coordinates (*M. marinum* M CDS locus tags)	Size (bp)	Description
MSRD7	175,088 - 180,287 (MMAR_RS00725-MMAR_RS00745)	5200	Contains 5 CDS, including an ATP-binding protein, membrane protein, glycosyltransferase, acyl-coA dehydrogenase, and transcriptional regulator<br>Flanked by ISMysh03
MSRD8	190,298 - 226,341 (MMAR_RS00790 – MMAR_RS00980	36044	Large RD, overlaps with MURD from MMAR_RS00915 – MMAR_RS00980. 7 additional mammalian cell entry proteins, 2 ABC transporters, and a Yrb integral membrane protein.<br>Flanked by ISMysh01
MSRD9	230,339 - 233,921 (MMAR_RS00990-MMAR_RS00995)	3583	Contains hypothetical proteins with annotated repeat units<br>Flanked by ISMysh01
MSRD13*	412,021 - 430,980 (MMAR_RS01785-MMAR_RS01815	18960	Includes non-ribosomal peptide synthetase CDS as well as three protein kinases and a thioesterase
MSRD14*	566,481 - 568,809 (MMAR_RS02365-MMAR_RS02375)	2329	Contains several hypothetical proteins
MSRD19*	1,126,581 - 1,127,535 (MMAR_RS04565)	955	Includes single cytochrome P450 CDS
MSRD26*	1,542,444 - 1,544,672 (MMAR_RS06255-MMAR_RS06260)	2229	Includes kinase and response regulator of two-component receptor
MSRD27	1,672,114 - 1,675,415 (MMAR_RS06805-MMAR_RS06815)	3302	Includes peptidyl-prolyl cis-trans isomerase and arylsulphatase CDS<br><br>Flanked by ISMysh01
MSRD31*	1,822,360 - 1,823,516 (MMAR_RS07455)	1161	Partial acetolactase synthase CDS
MSRD33	1,895,536 - 1,895,922 (MMAR_RS07780-MMAR_RS07790)	1906	Contains hypothetical proteins and putative hydrolase<br>Flanked by ISMysh01
MSRD34	1,901,816 - 1,908,672 (MMAR_RS07810-MMAR_RS07840)	6857	Includes several metabolic CDS, transcriptional regulator, and siderophore biosynthesis protein<br>Flanked by ISMysh03
MSRD35	1,929,502 - 1,971,741 (MMAR_RS07885-MMAR_RS08070	42240	Large RD, overlaps with MURD37 through MMAR_RS07920. Contains small (2783bp) RD from ATCC927. Region includes several CoA processing proteins, including dehydrogenases, ligases, and acyltransferases. Region also includes DNA ligase B, several transcriptional regulators, multicopper oxidase protein, and carbon starvation protein CstA<br><br>Flanked by composite ISMysh03/ISMysh01 locus

MSRD40*	2,520,457 - 2,521,761 (MMAR_RS10430)	1305	Hypothetical protein
MSRD47	2,947,071 - 2,947,848 (MMAR_RS12170)	778	Ketosteroid isomerase
MSRD48	2,974,005 - 2,982,746 (MMAR_RS12265- MMAR_RS12280)	8742	Hypothetical proteins<br>Flanked by ISMysh01
MSRD52*	3,102,829 - 3,117,465 (MMAR_RS12735- MMAR_RS12815	14637	Contains two small RD from ATCC927, galactose mutarotase, hydrolases, and a polyprenyl synthetase CDS, as well as several hypothetical proteins
MSRD55	3,207,968 - 3,209,243 (MMAR_RS13125)	1276	mannosyltransferase<br>Flanked by ISMysh01
MSRD56	3,228,171 - 3,232,355 (MMAR_RS13225- MMAR_RS13235)	4185	O-acyltransferase, hypothetical protein, transcriptional regulator<br>Flanked by ISMysh03
MSRD57	3,390,793 - 3,391,727 (MMAR_RS13895)	935	Hypothetical protein
MSRD58	3,405,689 - 3,407,973 (MMAR_RS13985)	2285	Hypothetical protein
MSRD64	3,699,478 - 3,709,392 (MMAR_RS15255- MMAR_RS15285)	9915	Hypothetical proteins, contains small (1354bp) RD from ATCC927<br>Flanked by multiple copies of ISMysh01, ISMysh03, ISMysh04
MSRD68	4,037,047 - 4,046,933 (MMAR_RS16315- MMAR_RS16365)	9887	Overlap with MURD68 in MMAR_RS16315, region includes hypothetical proteins, sigma factor CDS and acetolactose synthase.<br>Flanked by ISMysh03
MSRD76	4,342,991 - 4,346,022 (MMAR_RS17625- MMAR_RS17635)	3032	Hypothetical proteins and phenazine biosynthesis CDS<br>Flanked by multiple copies of ISMysh01
MSRD79	4,480,305 - 4,483,632 (MMAR_RS18125- MMAR_RS18135)	3328	Formate dehydrogenase and adenylate cyclase CDS<br>Flanked by ISMysh01
MSRD82	4,800,139 - 4,802,271 (MMAR_RS19285- MMAR_RS19290)	2133	YrbE family protein and ABC transporter permease<br>Flanked by ISMysh03
MSRD84	4,871,308 - 4,872,605 (MMAR_RS19760)	1298	Putative membrane protein
MSRD88	5,213,949 - 5,215,557 (MMAR_RS21205)	1609	ATPase<br>Flanked by ISMysh01
MSRD93	5,523,108 - 5,525,944 (MMAR_RS22535- MMAR_RS22550)	2837	Methyltransferase, hypothetical proteins<br>Flanked by ISMysh03
MSRD94	5,530,599 - 5,535,851 (MMAR_RS22570- MMAR_RS22590)	5253	Glycosyl and methyltransferases, hypothetical proteins<br>Flanked by ISMysh03
MSRD95	5,539,533 - 5,540,568 (MMAR_RS22610)	1036	Methyltransferase CDS<br>Flanked by ISMysh01
MSRD98	5,698,628 - 5,703,127	4500	Putative membrane protein, ferredoxin<br>Flanked by ISMysh03

	(MMAR_RS23280-MMAR_RS23290)		
MSRD102	6,083,337 - 6,084,477 (MMAR_RS251250-MMAR_RS251300)	1141	Oxidoreductase, ketosteroid isomerase
MSRD103	6,326,680 - 6,327,720 (MMAR_RS26285-MMAR_RS26290)	1041	Hypothetical proteins
MSRD105*	6,388,298 - 6,396,034 (MMAR_RS26565)	7737	PE-PGRS family protein

*Indicates *M. shottsii* regions of deletion also absent in *M. marinum* ATCC927, but present in *M. ulcerans*.

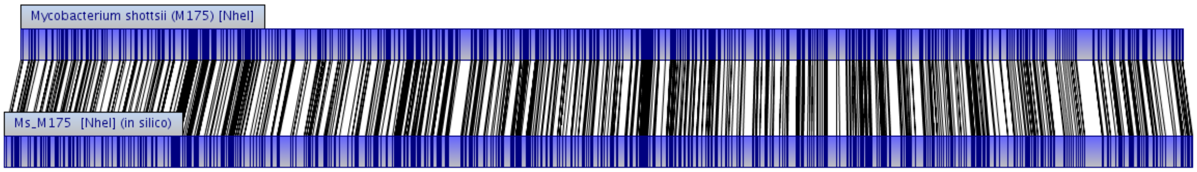

Supplemental Figure S1: Alignment of *M. shottsii* M175 genome maps from optical mapping versus PacBio sequencing. Using Mapsolver software, the *Nhe*I restriction site map of *M. shottsii* M175 generated by Opgen optical mapping (upper map) was aligned to the *in silico Nhe*I site mapping of the imported PacBio M175 genome sequence (lower map). Vertical blue lines within each map and black lines between genome maps indicate *Nhe*I sites. The alignment indicates no inversion differences between the maps. The optically-mapped genome size is smaller due to some restriction fragments less than 2 kb losing adherence to the support matrix prior to measuring fragments.
